# One-Step Purification of Recombinant Cutinase from an *E. coli* Extract Using a Stabilizing Triazine-Scaffolded Synthetic Affinity Ligand

**DOI:** 10.3390/biomimetics9010057

**Published:** 2024-01-20

**Authors:** Luís P. Fonseca, M. Ângela Taipa

**Affiliations:** 1 Department of Bioengineering, Instituto Superior Técnico, Avenida Rovisco Pais, 1049-001 Lisbon, Portugal; 2 Institute for Bioengineering and Biosciences (iBB), Institute for Health and Bioeconomy (i4HB), Instituto Superior Técnico, Avenida Rovisco Pais, 1049-001 Lisbon, Portugal

**Keywords:** recombinant cutinase, *E. coli*, affinity chromatography, biomimetic affinity ligands, triazine-scaffold, combinatorial library

## Abstract

Cutinase from *Fusarium solani pisi* is an enzyme that bridges functional properties between lipases and esterases, with applications in detergents, food processing, and the synthesis of fine chemicals. The purification procedure of recombinant cutinase from *E. coil* extracts is a well-established but time-consuming process, which involves a sequence of two anionic exchange chromatography steps followed by dialysis. Affinity chromatography is the most efficient method for protein purification, the major limitation of its use being often the availability of a ligand selective for a given target protein. Synthetic affinity ligands that specifically recognize certain sites on the surface of proteins are highly desirable for affinity processes due to their cost-effectiveness, durability, and reusability across multiple cycles. Additionally, these ligands establish moderate affinity interactions with the target protein, making it possible to purify proteins under gentle conditions while maintaining high levels of activity recovery. This study aimed to develop a new method for purifying cutinase, utilizing triazine-scaffolded biomimetic affinity ligands. These ligands were previously screened from a biased-combinatorial library to ensure their binding ability to cutinase without compromising its biological function. A lead ligand, designated as 11/3′, [4-({4-chloro-6-[(2-methylbutyl)amino]-1,3,5-triazin-2-yl}amino)benzoic acid], was chosen and directly synthesized onto agarose. Experiments conducted at different scales demonstrated that this ligand (with an affinity constant K_a_ ≈ 10^4^ M^−1^) exhibited selectivity towards cutinase, enabling the purification of the enzyme from an *E. coli* crude production medium in a single step. Under optimized conditions, the protein and activity yields reached 25% and 90%, respectively, with a resulting cutinase purity of 85%.

## 1. Introduction

Aerial plant organs are protected by a cuticle constituted by a polymeric structural compound, cutin, based on an insoluble lipid-polyester matrix composed mainly of hydroxy and hydroxy epoxy fatty acids covering the surface of plants [1]. Cutinases are enzymes named due to their ability to catalyze the breakdown of those polyesters and degrade cutin [2]. Cutinases were initially identified according to their biosynthesis by pathogenic fungi that participate actively in the invasion of host plants, although some bacteria produce them too. Cutinases are also biosynthesized by plants as these enzymes are probably involved in the synthesis of the lipid–polyester matrix that forms the cuticle [3]. In the realm of industrial applications, cutinases initially demonstrated remarkable efficiency in hydrolyzing a wide range of substrates such as esters, polyesters, and a large variety of short- and long-chain triacylglycerols and waxes. Subsequently, cutinases were explored by their reverse reaction, actively participating in catalyzing esterification, polymerization, and transesterification processes for the production of esters, polyesters, and novel triacylglycerols, among other compounds [4]. The high catalytic versatility of cutinases stems from a structural difference compared to classical lipases, as they do not exhibit interfacial activation, i.e., they do not present a hydrophobic lid covering the active site [5]. Additionally, their high flexibility allows easy access for a variety of short and long substrates, as well as solvents, facilitating the execution of multiple different reactions [6].

Cutinase from phytopathogenic fungus *Fusarium solani pisi* is a small carboxylic ester hydrolase that bridges functional properties between lipases and esterases. It belongs to the family of serine hydrolases, which are characterized by possessing an α/β hydrolase fold pattern. The active site, containing the catalytic triad Ser-120, Asp-175, His-188, is accessible to the solvent, which enables the enzyme adaptation to different substrates. [7,8,9]. *Fusarium solani pisi* cutinase was first cloned and expressed with success in *E. coli*, and its 3D structure was solved at 1.6 Å by Martinez et al. [2] and at 1.0 Å by Longhi et al. [6]. Cutinase is a compact one-domain molecule with 197 amino acids and a molecular weight of 22 kDa, presenting a hydrophobic core comprising a five-parallel-stranded β-sheet, surrounded by four α-helices [9]. It displays an innate ability to hydrolase lipids, catalyze esterification and transesterification reactions, and can be used as an efficient catalyst both in solution and at water-lipid interfaces [10]. Cutinase is therefore an enzyme of great interest for many industrial applications related to detergents (for lipid stain removal), food processing, the synthesis of fine chemicals, and the textile industries [3,11,12]. In recent years, cutinase from *F. solani pisi* has also gained particular attention as a promising candidate for the enzymatic depolymerization of poly-(ethylene terephthalate) (PET) and biocatalytic recycling plastic processes [13,14,15].

Recombinant cutinase has been produced and used over years in our lab as a model-enzyme for a variety of studies [16,17]. The protocol used for its purification is a well-established but time-consuming process that involves an osmotic shock, an acidic precipitation, and two anion exchange chromatographic steps, followed by dialysis [18].

Affinity chromatography is one of the most versatile and powerful techniques for purifying proteins from complex mixtures. Its operational concept relies on the reversible, specific binding occurring between an immobilized ligand and the product being purified. By optimizing binding and elution conditions, affinity chromatography enables the purification of a target protein or enzyme with high purity in a single-step process [19,20]. However, its use instead of classic chromatographic techniques is often limited by the availability of a cost-effective ligand recognizing the protein of interest. Affinity ligands for protein purification (as well as for other purposes that take advantage of affinity interactions) can be natural molecules, such as enzyme substrates and inhibitors, effectors, coenzymes, hormones, antigens, nucleic acids and sugars, for example, [21] or bioinspired affinity ligands [22] such as short mimetic peptides [23], aptamers [24], or rationally designed biomimetic triazine-scaffolded affinity ligands [25,26,27,28]. Triazine-based synthetic ligands are nontoxic, highly stable, cost-effective, low-molecular weight compounds that can replace advantageously natural biological ligands in the purification of proteins by affinity chromatographic methods [27,28]. These synthetic ligands mimic the structure and binding of natural ligands and are easily synthetized by a solid-phase assembly method on agarose matrix with a triazine scaffold. The triazine scaffold allows for the display of attached functional groups and the possibility of attaining molecular diversity by serial substitution of the chlorines in the triazine ring by commercially available aliphatic and aromatic amines [28].

Cutinase from *Fusarium solani pisi* was used as the model protein to develop an innovative methodology for assessing and enhancing protein stability. The exploitation of X-ray crystallographic data and cutinase surface using computer modelling tools allowed for the identification of target amino acid residues in three main regions where cutinase’s unfolding initiates. A 64-member solid-phase combinatorial library of *de novo* designed triazine-based bi-substituted complementary affinity ligands was synthesized and screened to bind cutinase in its active state [29]. The best binding substituents were then combined with those selected from the screening of a 164-member random library [30] to create a 36-membered second-generation biased library, following a semi-rational approach [31]. Ligands exhibiting protein yields above 50% when binding to pure cutinase while retaining substantial biological activity (≅50% of specific activity relative to the free enzyme) were utilized in conducting thermal stability assays with adsorbed or immobilized cutinase [31]. The primary goal of the present study was to evaluate some biomimetic affinity ligands, sourced from the biased-combinatorial solid-phase library, potentially capable of targeting a distinct site on cutinase’s surface, to establish a novel, more effective purification process using affinity chromatography.

## 2. Materials and Methods

### 2.1. Reagents

Sucrose, Tris-HCl, EDTA, NaOH, HCl, NaCl, isopropanol, glycine, cyanuric acid, 2,4,6-trinitrobenzenesulphonic acid (TNBS), 2-methylbutylamine, 4-aminobenzoic acid, sodium citrate, bicinchoninic acid (BCA), bovine serum albumin (BSA), *p*-nitrophenylbutyrate (*p*-NPB), acetonitrile, Sodium dodecyl sulfate, acrylamide and bia-acrylamide, ethanol, acetic acid, sodium thiosulfate, silver nitrate, formaldehyde, and sodium carbonate were purchased from Sigma-Aldrich (Darmstadt, Germany). BCA^TM^ (bicinchoninic acid) and Micro BCA^TM^ Protein Assays Reagents were from ThermoScientific (Rockford, IL, USA). Sepharose CL-6B was purchased from Sigma-Aldrich. The anion-exchange chromatography (DEAE-cellulose and Q-Sepharose) columns used were from GE Healthcare (Amersham, UK).

### 2.2. Production and Conventional Purification Process of Cutinase

Recombinant cutinase from *F. solani pisi* was cloned in pMa5-L [32] and over expressed in *Escherichia coli* WK-6. The enzyme was produced and purified according to a protocol developed and implemented at our lab [33]. After centrifugation, cutinase was recovered from the periplasmic space by osmotic shock. Osmotic shock was divided in four steps; first the cells were resuspended in TE buffer (EDTA, 12.5 mM, in 1 M Tris-HCl, pH 8), then centrifuged and resuspended in ST buffer (sucrose, 20% (*w*/*v*), in 1 M Tris-HCl, pH 8.0). The process was repeated for STE buffer (sucrose, Tris-HCl, EDTA) and cold water. The enzyme extract was further submitted to acid precipitation (overnight) with acetic acid 1:2 (*v*/*v*) at pH 4.7 at 4 °C. After the acid precipitation step, a centrifugation was performed and the supernatant was subjected to a first dialysis step (48 h) using cellulose tubing, and placed in 5 L of buffer (Tris-HCl, 20 mM, pH 7.6) at 4 °C, with mild magnetic stirring. The buffer was exchanged three times over the 48 h. The dialysis sample was purified by two sequential anion-exchange chromatographic steps, using DEAE-cellulose and Q-Sepharose HP columns, in an ÄKTApurifier 100 system (GE, Amersham, UK). The fraction containing the pure cutinase was then dialyzed for 24 h in distilled cold water at 4 °C. The two anion-exchange chromatographic steps and the final dialysis are herein termed ‘conventional purification process’ in order to compare them with the novel affinity chromatography process developed for cutinase purification.

### 2.3. Screening of Biomimetic Affinity Ligands via Affinity Chromatographic Assays

Triazine-scaffolded affinity ligands from a second-generation combinatorial library synthesized by Sousa et al. [30] were screened and tested, at a bench-scale, for cutinase purification. The affinity columns contained 1 mL of moist gel, packed into 4 mL columns. Each column was connected to a peristaltic pump in order to maintain a constant flow rate of 1 mL/min. The column was first washed with 15 column volumes (CVs) of regeneration solution (0.1 M NaOH in 30% (*v*/*v*) isopropanol) and with 15 CVs of distilled water to bring the pH to neutral. The resin was then equilibrated with 25 CVs of equilibration buffer. Samples of 15 mL of first dialysis extracts (impure extracts) were applied to the columns. After sample application, the column was washed with equilibration buffer to remove unbound proteins. Fractions of 1 mL were collected until the absorbance at 280 nm reached a value equal or inferior to 0.005 (about 30 CVs), and cutinase activity was measured in every sample. These fractions were further pooled in order to form the ‘washing pool’ sample. Bound cutinase was eluted with 100 mM glycine-HCl, pH 2.0 (selected from previous studies, unpublished data), and 1 mL fractions were collected and neutralized immediately with 150 µL of 1 M Tris-HCl, pH 9.0.

The elution step was performed using different CVs, ranging from 25 to 35. The collected fractions that corresponded to the first elution peak were pooled to form the ‘elution peak’ sample, while the remaining (containing traces of enzymatic activity) were also pooled and formed the ‘elution pool’ sample. After elution, the column was washed with 15 CVs of regeneration solution, followed by 15 CVs of distilled water, and stored in 20% (*v*/*v*) ethanol at 4 °C. From the screening assays, ligand 11/3′ was selected as a lead for further studies and was *de novo* synthesized, in a high amount, by solid-phase synthesis.

### 2.4. Solid-Phase Synthesis of Ligand 11/3′

Ligand 11/3′ was synthesized by a well-established solid-phase synthesis protocol, as described in [28]. Sepharose CL-6B (support matrix) was activated with epichlorohydrin, followed by amination and reaction with cyanuric chloride. The cyanuric chloride-activated gel (dichlorotriazinyl agarose) was immediately used for the substitution of the two remaining chlorine atoms, designated as R1 and R2. The R1 and R2 reactive chlorines on the triazine scaffold were sequentially substituted with selected amines, mimicking the side chains of specific amino acids.

The density of primary amine groups on aminated Sepharose beads was determined by a 2,4,6-trinitrobenzenesulphonic acid (TNBS)-based method, as described by Antoni et al. [34]. The aminated support used for activation with cyanuric chloride presented a value of 19.5 µmol amine groups/g moist weight gel. The cyanuric-activated gel was used for chlorine substitutions, under controlled reaction conditions. For R1 substitution the gel was incubated with 2-methylbutylamine, at 30 °C for 24 h, in a rotary shaker. The mono-substituted R1 gel was then incubated with 4-aminobenzoic acid, at 85 °C, for 72 h, in a rotary oven, for R2 substitution. This procedure resulted in the synthesis of [4-({4-chloro-6-[(2-methylbutyl)amino]-1,3,5-triazin-2-yl}amino)benzoic acid] (ligand 11/3′).

#### 2.4.1. Purification of Cutinase by using Affinity Chromatography with Lead Ligand 11/3′

The newly synthesized ligand-adsorbent 11/3′ was first tested in a 1 mL column using the method described above (Section 2.3), in order to ensure that it continued to exhibit selectivity for cutinase. A volume of 15 mL of derivatized Sepharose CL-6B (bed height of 7.5 cm) with the *de novo* synthesized ligand was packed into a HiScale^TM^ 16/20 column (GE Healthcare, Amersham, UK). The column was connected to an ÄKTApurifier 100 (GE, Amersham, UK) system and operated at a flow rate of 15 mL/min. The sample loaded onto the column was 225 mL of impure extract, obtained after the first dialysis step (Section 2.2). The column was washed and equilibrated with the same CVs than in the assays performed with the 1 mL column (Section 2.3). Regarding the elution step, 35 CVs of 100 mM M glycine-HCl, pH 2.0, were used, and 12 mL samples were collected using a Frac-900 (GE Healthcare, Amersham, UK) collector. The elution samples were immediately neutralized with 1.8 mL of 1 M Tris-HCl, pH 9.

#### 2.4.2. Elution Assays for Optimizing Cutinase Purification

Elution assays with different buffers were performed in a column containing the *de novo* synthesized lead ligand 11/3′. The derivatized resin was packed into a 5/100 TricornTM high-performance column (GE Healthcare, Amersham). A total of 1.7 mL of moist gel (bed height of 87 mm) was used. The column was connected to a ÄKTApurifier 100 (GE, Amersham). Column regeneration, rinsing using distilled water, and equilibration followed the same procedures with column volumes (CVs) as those employed in earlier tests. A flow rate of 3 mL/min was maintained during these stages, whereas sample loading and elution steps utilized a flow rate of 1.7 mL/min. A sample of 20 mL of the first dialysis extract was loaded onto the column. Different elution buffers were assayed with the aim of improving cutinase elution from the column. The elution buffers under test were labeled sequentially by numbers (1 to 6): elution buffer 1 (100 mM glycine-HCl, pH 2.0), used in the previous elution assays, was considered the control buffer; elution buffer 2 (250 mM glycine-HCl, pH 2.0); elution buffer 3 (100 mM M glycine-HCl with 0.1 M NaCl, pH 2.0); elution buffer 4 (5 mM citrate buffer, pH 3.0); elution buffer 5 (10 mM citrate buffer, pH 3.0), and elution buffer 6 (5 mM citrate buffer, pH 4.0). Twenty CVs were used for the elution step, and fractions of 2 mL were collected. Neutralization to pH 8.0 of all the samples collected during elution was performed immediately after collection, with an adjusted volume of neutralizing solution, according with the elution buffer tested.

### 2.5. Protein Quantification Assays

The quantification of total protein was performed with the bicinchoninic acid (BCA) method [35]. A volume of 25 µL of sample solution was added to 200 µL of Pierce reagents (50:1 of reagent A in B) in a 96-well microplate. After mixture, the microplate was incubated for 30 min at 37 °C and absorbance was read at 562nm in a microplate reader from Molecular Devices (Sunnyvale, CA, USA). A calibration curve was prepared with bovine serum albumin (BSA), within a concentration range from 0 µg/mL to 2000 µg/mL. Each sample was analyzed in triplicate. The Micro BCATM Protein Assay (for a protein concentration range from 2 µg/mL to 40 µg/mL) was also used, when necessary, following the instructions provided by the manufacturer.

### 2.6. Enzyme Activity Assay

Cutinase activity was determined spectrophotometrically, following the hydrolysis of *p*-nitrophenylbutyrate (*p*-NPB) at 400 nm (ε = 18,400 M^−1^cm^−1^) [36]. A stock solution of 70 mM *p*-NPB was prepared in pure acetonitrile. Activity assays were performed with addition of 15 µL of sample to 1470 µL of 20 mM Tris-HCl buffer, pH 8.0, in a stirred 4 mL cuvette, at 30 °C. The activity assay was initiated by adding 15 µL of *p*-NPB stock solution. Release of *p*-nitrophenol was monitored by reading the absorbance at 400 nm every 10 s for one minute, against a blank containing 20 mM Tris-HCl buffer, pH 8.0, using a Hitachi U-2000 spectrophotometer (Hitachi, Tokyo, Japan). One unit of activity was defined as the amount of enzyme required to convert 1 µmol of *p*-NPB to *p*-nitrophenol per minute under standard assay conditions.

### 2.7. SDS-PAGE Gel Electrophoresis

Sodium dodecyl sulfate polyacrylamide gel electrophoresis (SDS-PAGE) analysis was performed for protein characterization. Resolving and stacking gels were prepared according to a protocol described in [37].

Gels were polymerized in properly assembled Bio-Rad (Hercules, CA, USA) cassettes. Samples obtained in different experiments performed were submitted to a concentration step by adding 3 mL of sample Amicon^®^ Ultra-4 mL centrifugal filter units (Merck Millipore, Burlington, MA, USA), with a molecular weight cut-off of 10 kDa. The tubes were centrifuged at 4.4 × 1000× *g* during 45 min. Protein concentration was determined after centrifugation with the BCATM assay. Electrophoresis was run at 90 V for 2 h. Gels were stained using the silver-staining method. The gels were placed sequentially in the following solutions: fixation solution for 1 h (50% (*v*/*v*) ethanol; 12% (*v*/*v*) acetic acid in Milli-Q water); 3 × 20 min in ethanol 30% (*v*/*v*); oxidizing solution (0.8 mM sodium thiosulphate) for 10 min; fresh silver nitrate solution (11.8 mM silver nitrate, 0.02% formaldehyde in Milli-Q water) for 30 min; 3 × 1 min in Milli-Q water; developing solution (0.566 M sodium carbonate, 0.02 M sodium thiosulphate, 0.02% formaldehyde in Milli-Q water) until the solution turned yellow and the protein bands appeared; and finally in fixation solution again for 15 min. The purity of cutinase in silver-stained gels was estimated using the MyImageAnalysis software, version 1.1 (Thermo Scientific, Waltham, MA, USA). The intensities of protein bands were quantified by densitometry analysis and the purity of cutinase was calculated by the ratio between the intensity of the cutinase band and the intensities of all protein bands present in the sample.

## 3. Results and Discussion

### 3.1. Screening of Affinity Ligands from a Solid-Phase Combinatorial Library

Several ligands from a solid-phase synthetic ligand library (Table 1) were screened for the selective binding and purification of cutinase from *E. coli* crude extracts by affinity chromatographic assays on a bench scale (1 mL moist gel), using 100 mM glycine-HCl, pH 2.0, as the elution buffer.

The synthetic affinity ligands were divided according to the R1 and R2 substituents: ligands with one charged and one hydrophobic substituent (ligand 11/3′, ligand 3′/11, ligand 5/3′, ligand 3′/5, ligand 3/3′, ligand 4/3′) and ligands with two hydrophobic substituents (ligand 3′/3′ and ligand 6/3′). In previous studies, ligands with one negatively charged and one hydrophobic moiety have been shown to stabilize bound cutinase when compared with the free enzyme, while ligands with two hydrophobic substituents exhibited a destabilizing effect on the bound enzyme [30]. In all tested ligands, the breakthrough fraction and washing pool showed negligible cutinase activity, measuring below 0.5 U/mL. Yet, for most ligands, SDS-PAGE gel analysis verified the existence of residual cutinase in these pools. Despite this, the samples did not exhibit measurable cutinase activity. It was anticipated that cutinase would bind selectively to the synthetic ligands, being recovered only in the elution step. However, a slight likelihood of cutinase exclusion might be somehow expected, since ligand adsorbents with comparable ligand density (approximately 20 μmol ligand/g moist gel) had previously demonstrated a binding capacity of ca. 90% for pure cutinase [31].

For most of the ligands assessed, a main elution peak with cutinase activity was obtained, followed by a more or less significant dragging in the elution of enzymatic activity. The pool of these last fractions was designated the ‘elution pool’ (as to distinguish from the main concentrated ‘elution peak’). The elution buffer selected plays, likely, a key role in the described elution profile, due to its inability to break completely the non-covalent interactions formed between the cutinase surface and the synthetic affinity ligands, hence causing a dragging effect in the elution profile.

**Figure 1:**
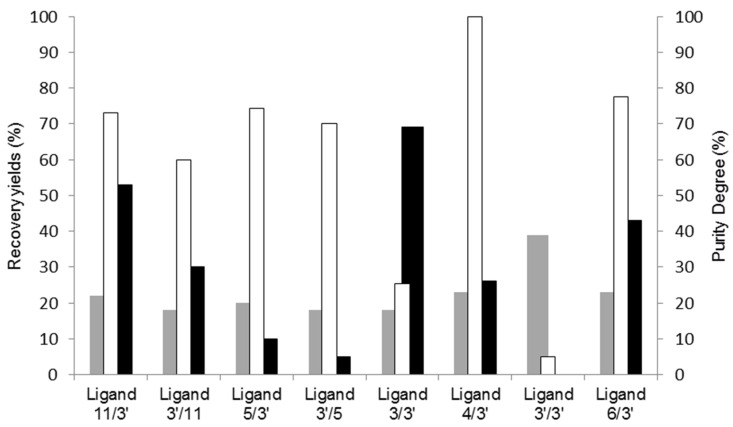
Screening of synthetic affinity ligands from a combinatorial library for cutinase purification by affinity chromatography. Recovered protein yields in the elution step (grey), activity yields at the elution step (black), and cutinase purity degree (no fill) at the main elution peak are represented for the affinity chromatographic assays performed with all the solid-phase screened ligands.

A summary of the results obtained for the various ligands screened is depicted in Figure 1.

Upon examining Figure 1, it becomes evident that solely two ligands (11/3′ and 3/3′) exhibited an activity recovery yield surpassing 50%. However, ligand 11/3′ enabled a higher purity degree of recovered cutinase by SDS-PAGE analysis when compared to 3/3′. Ligand 4/3′ yielded the highest cutinase purity, whereas ligand 3′/3′ failed to selectively capture cutinase from the crude extract.

Based on these preliminary studies conducted under non-optimized conditions, ligand 11/3′ was chosen as the lead candidate to evaluate the scalability of affinity chromatography using biomimetic affinity ligands for cutinase purification. This decision factored in the comprehensive performance evaluation of the tested ligands, including their elution profiles, yields, and specificity toward cutinase. Ligand 11/3′ was previously selected as one leading to the highest activity retention of bound cutinase. Solid-phase synthesized ligand 11/3′ enhanced considerably the activity retention of adsorbed cutinase at high temperatures (60–80 °C), relative to the free enzyme [31].

### 3.2. Cutinase Purification Using Affinity Chromatography with Ligand 11/3′

Ligand 11/3′ was *de novo* synthesized by solid-phase synthesis [26,27]. Before testing it at a larger scale, affinity chromatographic assays with a column containing 1 mL of moist gel were performed, in triplicate, as a control. The *de novo* synthetized ligand 11/3′ showed identical selectivity toward cutinase, enabling its elution and purification from an impure first dialysis extract. A dragged cutinase activity elution was also verified after the main elution peak. The global evaluation of this chromatography assay is described in Table 2.

In order to evaluate the elution step for this chromatographic process, the ‘elution peak’ and ‘elution pool’ were pooled together, forming the ‘total elution pool’. The elution pool presents a more significant protein yield than the elution peak. However, the activity yields were similar. Altogether, the newly synthesized ligand enabled a protein recovery of 92% and an activity recovery of 77% throughout the entire chromatographic procedure. In the total elution pool, a purification factor of 4.2 (relative to the first dialysis extract) was estimated, with an activity recovery yield of 102%. This yield may be due to the possible overestimation of cutinase activity in this sample. Or, oppositely, the disparity in the sum of the activity yields of the elution peak and pool (77%) against the one obtained in the total elution pool (102%) may be explained by an underestimation of enzyme activity in the elution peak.

**Figure 2:**
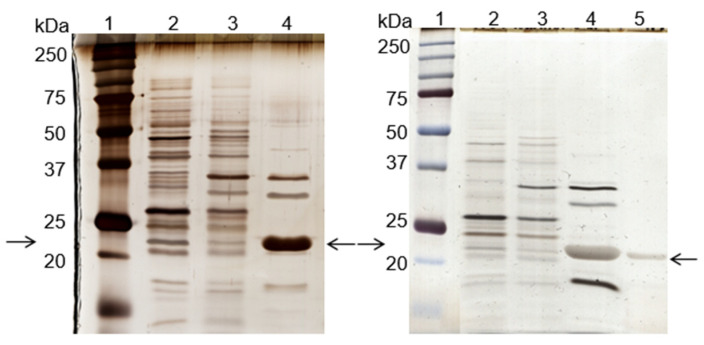
Silver-stained SDS-PAGE gels of the control (**left**) and scale-up (**right**) experiments, using the *de novo* synthetized solid-phase ligand 11/3′ for cutinase purification by affinity chromatography. Each well was loaded with 1 µg of total protein. On the left: lane 1, molecular weight marker; lane 2, breakthrough; lane 3, washing pool; lane 4, total elution pool (elution peak + elution pool). On the right: lane 1, molecular weight marker; lane 2, breakthrough; lane 3, washing pool; lane 4, elution peak; lane 5, elution pool. Arrows indicate the position of the protein band corresponding to cutinase.

Two additional experiments were performed, under the same conditions, with the *de novo* synthetized 11/3′ ligand. The total protein recovery yield of the process in all experiments was above 90%, indicating that only a minor part of the total protein loaded onto the column remained strongly bound. The overall performance, taking into account three experiments performed, can be summarized by a purification factor of 6.8 ± 1.2 and 1.9 ± 0.3 for the elution peak and elution pool, respectively. The specific activity of the elution peak achieved a value of 1313 ± 241 U/mg protein. Protein yield varied between 3% and 7%, while activity yield ranged from 26% to 38% at the elution peak. Analysis by SDS-PAGE (Figure 2, left gel) confirmed that the ligand bound specifically to cutinase although the non-specific adsorption of few contaminant proteins was still observed. A cutinase purity degree of 57% was estimated for cutinase in the total elution pool.

**Figure 3:**
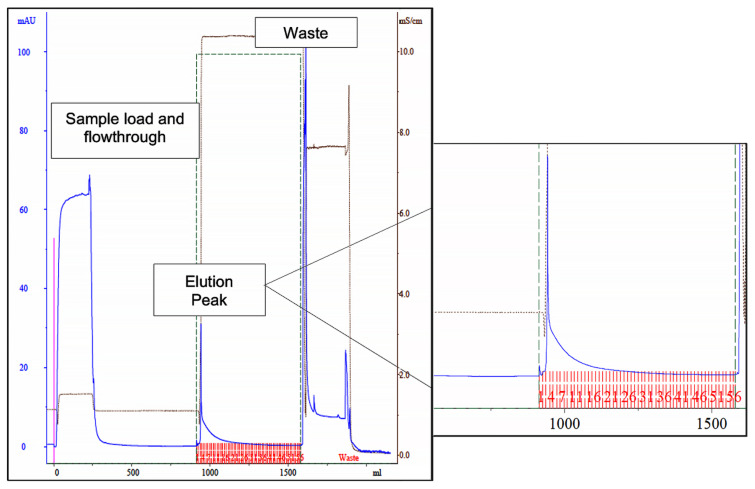
Chromatogram of the scale-up assay, using newly synthetized ligand 11/3′ for cutinase purification by affinity chromatography. Absorbance at 280 nm was measured for protein quantification (blue line). The elution peak is highlighted on the right, denoting a dragged protein elution after the elution peak. The fractions collected during elution are represented in red in the highlighted section. The waste corresponds to column regeneration with 0.1 M NaOH, 30% (*v*/*v*) isopropanol.

A column containing 15 mL of *de novo* synthetized ligand 11/3′ was then packed and used in a larger scale process. The chromatogram (Figure 3), yields and purification factor obtained in two experiments performed with at a larger scale were similar and comparable to the ones obtained in the control assay performed on a small scale with 1 mL of moist resin.

Through SDS-PAGE gel analysis (depicted in Figure 2, right gel), it was confirmed that cutinase was selectively bound in the process. However, the extent of non-specific adsorption of other contaminant proteins seemed to have increased in a larger scale, resulting in a cutinase purity of only 34%. Despite this, the results indicated the potential viability of using triazine-scaffolded ligand 11/3′ for purifying cutinase from impure extracts at a larger scale by affinity chromatography. Yet, the optimization of the conditions remained necessary. Throughout the conducted affinity chromatographic experiments, it became evident that the elution buffer used demonstrated an inherent inefficiency, impacting cutinase elution from the column as the enzyme activity was not totally concentrated in the elution peak. The subsequent section outlines experiments conducted with various elution buffers aimed at enhancing cutinase’s elution profile and efficiency.

### 3.3. Assessment of Different Elution Buffers to Enhance Cutinase Purification

**Figure 4:**
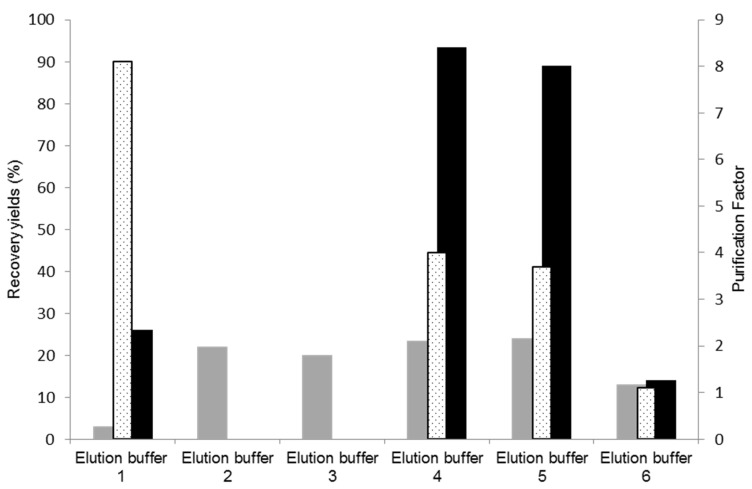
Assessment of different elution buffers for improvement of cutinase elution and purification. Protein yields (grey), activity yields (black), and purification factors (dotted) obtained at the main elution peak with the different elution buffers tested for cutinase purification by affinity chromatography with the *de novo* synthetized ligand 11/3′. The control corresponds to elution buffer 1.

Cutinase elution from a 11/3′ ligand-derivatized resin was tested with different buffers, with the aim of increasing selectivity and activity yield in the elution step. The results obtained are represented in Figure 4. The control experiment corresponds to use of the elution buffer 1 (100 mM glycine-HCl, pH 2.0). In these conditions, only 26% of activity was recovered in the main elution peak with a protein yield 3%. Elution buffers 2 (250 mM glycine-HCl, pH 2.0) and 3 (100 mM M glycine-HCl with 100 mM NaCl, pH 2.0), which result from slight modifications of buffer 1, were ineffective for eluting cutinase.

Elution buffer 4 (5 mM citrate buffer, pH 3.0) displayed enhanced effectiveness in the elution process, resulting in an activity yield of 90% in the main elution peak, a protein yield of 25%, and a purification factor of 4.0. These results are comparable to those detailed in Table 2, from a preliminary bench assay on a smaller scale, for ‘the total elution pool’, which comprised both the ‘main elution peak’ and the ‘pool of the elution step’ (dragging elution) utilizing elution buffer 1 (100 mM Glycine HCl). Consequently, employing buffer 4 (5 mM citrate buffer, pH 3.0) for elution did not lead to cutinase dilution/dragging, as 90% of the cutinase in the impure extract was concentrated and collected at the main elution peak. Although a trace contaminant still co-eluted with the target enzyme, citrate has demonstrated a stronger ability to disrupt non-covalent bonds established between cutinase and ligand 11/3′ compared to glycineHCl. Buffers 4 (5 mM citrate buffer, pH 3.0) and 5 (10 mM citrate buffer, pH 3.0) yielded similar results, while buffer 6 (similar to buffer 4 but with pH 4.0) did not elute cutinase from the resin.

**Figure 5:**
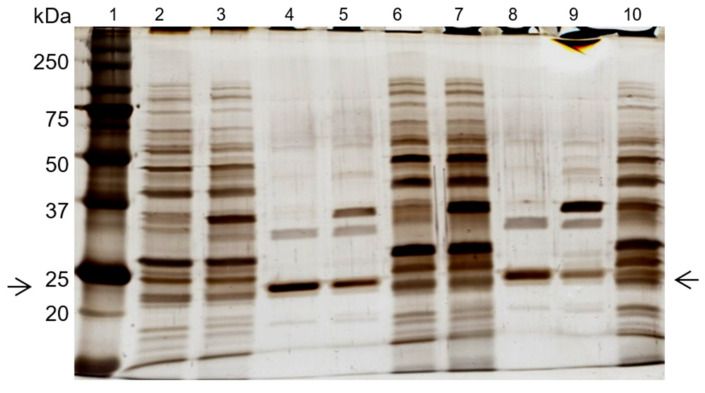
SDS-PAGE analysis of the effect of elution buffers 4 and 5 on cutinase purification, with the *de novo* synthetized ligand 11/3′. Each well was loaded with 1 µg of total protein. Lanes 2–5 represent the affinity chromatographic process with elution buffer 4. Lanes 6–9 correspond to the process with elution buffer 5. Lane 1, molecular weight marker. Lane 2, breakthrough. Lane 3, washing pool. Lane 4, elution peak. Lane 5, elution pool. Lane 6, breakthrough. Lane 7, washing pool. Lane 8, elution peak. Lane 9, elution pool. Lane 10, first dialysis extract. Arrows indicate the position of the protein band corresponding to cutinase.

SDS-PAGE gel analysis (Figure 5) confirmed cutinase purification in the elution step, with the combined use of newly synthetized ligand 11/3′ with elution buffers 4 and 5. The cutinase purity degree achieved with elution buffer 4 in the main elution peak was 85% (Figure 5, lane 4). This value is significantly higher when compared with the one obtained for the ‘total elution pool‘ (57%) using 100 mM Glycine HCl pH 2.0 as the elution buffer. For buffer 5, a purity degree of 63% was estimated for cutinase in the main elution peak (Figure 5, lane 8).

While a slight contaminant still co-eluted within the main elution peak, citrate exhibited a more robust capability in disrupting the non-covalent bonds formed between cutinase and ligand 11/3′ compared to glycine. Further experiments with a larger column volume and subtle adjustments to the citrate buffer are anticipated to eliminate completely protein contaminants from the elution peak, aiming for a purity of 100% in purified cutinase.

## 4. Conclusions

One-step affinity chromatography utilizing a dipeptide-mimic triazine-scaffold synthetic affinity ligand (11/3′) was shown to represent a viable, more cost-effective and less time-consuming alternative to the conventional purification method of recombinant cutinase from *Fusarium solani pisi.* Ligand 11/3′, which was formerly demonstrated to have a thermal stabilizing effect on cutinase, exhibited high selectivity in capturing the enzyme from *Escherichia coli* WK-6 crude extracts. Fine-tuning the elution process revealed that employing a 5 mM citrate buffer at pH 3.0 enables the retrieval of 90% of the total enzymatic activity in the main elution peak while achieving an 85% purity level for the purified enzyme. It is conceivable that minor adjustments to this buffer might yield similar recovery yields and ensure 100% purity for cutinase. As a generic strategy, selective biomimetic affinity adsorbents that simultaneously have a thermal stabilizing effect may be useful either for the purification of proteins or for the utilization of the macromolecular ligand–protein supports (e.g., as biocatalysts) under unfavorable conditions.

## Figures and Tables

**Table 1:** General structure of solid-phase synthetic ligands screened for the selective binding and purification of cutinase from *E. coli* crude extracts. The ligands display two substituents groups at the R1 and R2 positions. Aminated compounds, mimicking the side chains of specific amino acids, which were used for sequential substitution of the two reactive chlorines on the triazine scaffold, generating ligands 3/3′, 3′/3′, 4/3′, 3′/5, 5/3′, 6/3′, 3′/11 and 11/3′.

General Structure of the Biomimetic Adsorbents	Number	Aminated Compound R1 or R2	Analogue Amino Acids
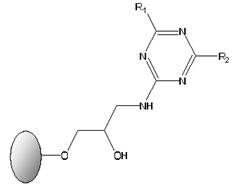	3 5 11 3′ 4′ 6	Tyramine Isoamlylamine 2-Methylbutylamine 4-Aminobenzoic acid 4-Aminophenylacetic acid 4-Aminobutyric acid	Tyrosine Leucine Isoleucine Aspartic acid Glutamic acid Aspartic acid Glutamic acid Glutamic acid

**Table 2:** Global evaluation of the affinity chromatographic assay, with the *de novo* synthetized ligand 11/3′ for cutinase purification, from 15 mL of extract after the first dialysis extract, in 1 mL moist-gel column. The yields and purification factors of subsequent steps were calculated by taking the first dialysis extract as the reference (activity yield = 100%, purification factor = 1).

Sample	Volume (L)	Protein (mg/L)	Total Protein (mg)	Activity (U/mL)	Total Activity (U)	Specific Activity (U/mg Protein)	Protein Yield (%)	Activity Yield (%)	Purification Factor
First dialysis extract	0.015	255.3	3.8	49.7	745.5	195	100	100	1.0
Breakthrough	0.015	100	1.5	0.3	4.5	3	39	1	0.0
Washing pool	0.04	27.0	1.1	0.0	0.0	0	28	0	0.0
Elution Peak	0.005	45.0	0.2	56.7	283.5	1260	6	38	6.5
Elution Pool ^1^	0.030	23.5	0.7	9.7	291.0	413	18	39	2.1
Total Elution Pool ^2^	0.035	26.8	0.9	21.8	763.0	813	24	≅100	4.2

^1^ Elution pool comprises elution fractions collected after the elution peak that presented cutinase activity. ^2^ Total elution pool results from the combination of the elution peak and elution pool.

## Data Availability

Data are included within this article.
